# Towards Caring Technologies in Older Adult Care Through the Co-Creation of an Ethical Process Guide

**DOI:** 10.3390/ijerph23020238

**Published:** 2026-02-13

**Authors:** Elisabeth Honinx, Cato van Schyndel, Arend Roos, Emily Paulding, Toni Wright, Kathleen Galvin, Theofanis Fotis, Jorg Huber, Erik Laes, Nathalie Lambrechts

**Affiliations:** 1 Vlaamse Instelling voor Technologisch Onderzoek (VITO), 2400 Mol, Belgium; erik.laes@vito.be (E.L.); nathalie.lambrechts@vito.be (N.L.); 2 Centre for Sociological Research, Faculty of Social Sciences, KU Leuven, 3000 Leuven, Belgium; 3 DigiRehab Nederland, 4334 EH Middelburg, The Netherlands; arend@roos.health; 4 Kent County Council, West Malling ME19 4AE, UK; emily.paulding@kent.gov.uk; 5 School of Nursing, Midwifery, Allied and Public Health, Canterbury Christ Church University, Canterbury CT1 1QU, UK; toni.wright@canterbury.ac.uk; 6 School of Education, Sports & Health Sciences, University of Brighton, Brighton BN1 9PH, UK; k.galvin@brighton.ac.uk (K.G.); t.fotis@brighton.ac.uk (T.F.); j.huber@brighton.ac.uk (J.H.)

**Keywords:** caring technology, digital health, process guide, responsible innovation, older adult care

## Abstract

**Highlights:**

**Public health relevance—How does this work relate to a public health issue?**

- Ageing populations and care workforce shortages are driving the rapid adoption of caring technologies in older adult care, often without sufficient ethical guidance to ensure inclusion, autonomy, and equity.
- Existing ethical frameworks in digital health remain largely abstract, which limits their practical use in public health and care settings.

**Public health significance—Why is this work of significance to public health?**

- This study provides an empirically grounded blueprint that translates ethical principles into practical guidance for caring technology development and implementation.
- By focusing on older adults as a vulnerable population, the study supports public health goals related to digital inclusion, equitable access, and mental well-being.

**Public health implications—What are the key implications or messages for practitioners, policy makers and/or researchers in public health?**

- Public health practitioners and innovation managers can use process-based ethical guidance to support responsible and inclusive technology adoption in care settings.
- Policymakers and researchers are encouraged to further validate and embed ethical process guides within innovation governance and public health evaluation frameworks.

**Abstract:**

As populations age, the gap between care needs and available support systems is widening, leading to critical vulnerabilities in staffing, infrastructure, and funding. The need for accessible, human-centred, and ethically grounded care technologies is growing. However, the development of digital health tools often lacks inclusivity and practical guidance. Existing ethical frameworks tend to remain abstract, which limits their real-world application. This study examines how such frameworks support the responsible development and implementation of caring technologies in older adult care. To achieve this, in-depth interviews were conducted with care providers, technology developers, and policymakers from partner organisations of the EMPOWERCARE project in the four participating countries: the UK, the Netherlands, Belgium and France. A core challenge was the limited applicability of abstract ethical principles in daily care settings. While existing initiatives often define ethical domains, few offer a structured, actionable process to guide implementation in practice. The proposed guide responds with a step-by-step structure, practical examples, and participatory tools to support inclusive, value-driven technology adoption. It is envisioned both as an implementation aid and a quality label to align stakeholders. Future research should validate the guide’s usability, explore its role across care contexts, and examine how ethics can be more firmly embedded in innovation governance.

## 1. Introduction

Population ageing is a global phenomenon that affects individuals, societies, and healthcare systems. Advances in medicine and public health have significantly increased life expectancy, leading to a growing proportion of older adults worldwide [1,2]. By 2050, the number of individuals aged 60 and above is projected to nearly double [3]. While this demographic transition reflects societal progress through increased life expectancy and improved living standards, it also brings about substantial challenges. Older adults are more likely to experience chronic illness, cognitive decline, and reduced mobility [3,4,5,6,7,8,9,10,11]. Combined with factors like social isolation and loneliness, this can seriously impact physical and mental wellbeing [12,13,14,15]. This results in rising demands for healthcare services and a widening gap between care needs and available support systems [9,16,17,18]. The COVID-19 pandemic has exacerbated these pressures, revealing critical vulnerabilities in staffing, infrastructure, and funding. The need for accessible, human-centred, and sustainable care becomes increasingly urgent. While digital health innovations are widely promoted as part of the solution, the real-world implementation of these technologies remains fragmented, often failing to reach those most in need [19,20,21,22,23,24,25,26,27]. In particular, older adults with limited digital literacy, mobility constraints, or socioeconomic disadvantages are at risk of being excluded from technological advances meant to support their health, independence, and societal participation [26,27].

Moreover, digital technology is not a silver bullet. Ethical concerns such as loss of autonomy, data privacy, and depersonalisation of care persist [28]. Current innovation ecosystems tend to prioritise efficiency and market scalability over emotional wellbeing or relational quality, leading to so-called “cold” technologies—tools that monitor or automate but do not meaningfully connect with older users’ lived realities [23,24,25,26,27]. If left unaddressed, these dynamics risk widening existing health and social inequalities rather than reducing them.

Policy frameworks also need to evolve. While healthcare technologies are subject to regulatory frameworks—such as CE marking in the EU or regional initiatives like the mHealth programme in Flanders—these standards primarily focus on safety, quality, and scientific validation of health outcomes [29,30,31,32,33,34]. Ethical and human-centred aspects, including usability, inclusivity, and alignment with end-users’ values and needs, are often addressed through non-binding guidelines or “soft law” initiatives, such as the European Ethical Principles for Digital Health or the WHO’s global digital health strategy [35]. However, such principles are not yet systematically embedded in regulatory processes, leaving a gap between technological validation and meaningful, user-centred implementation [28,36,37]. Addressing these structural issues is essential for achieving digital inclusion in ageing societies.

To date, Health Technology Assessment (HTA) is a well-established method for evaluating the effectiveness, safety, and cost-efficiency of healthcare innovations. However, it rarely addresses the broader social, ethical, or user experience dimensions that determine whether a technology is truly “caring” [23]. Several ethical frameworks have attempted to guide responsible technology development, including ethical technology assessment (eTA), value-sensitive design (VSD), and responsible research and innovation (RRI) [38]. Yet many of these remain high-level and are less able to set priorities or make normative decisions, offering limited practical utility to those tasked with real-world implementation [38]. This creates a vacuum between formal validation processes and the actual values and expectations of users, carers, and communities.

In response, a shift toward “caring technologies” is emerging [2]. These technologies are developed through participatory, interdisciplinary processes and aim to enhance autonomy, inclusion, and human-centeredness. They support not only physical health but also emotional resilience and social integration, central pillars of public mental health promotion. However, designing and implementing such technologies at scale requires more than good intentions: it calls for structured guidance, normative alignment, and inclusive decision-making processes [38].

**Figure 1:**
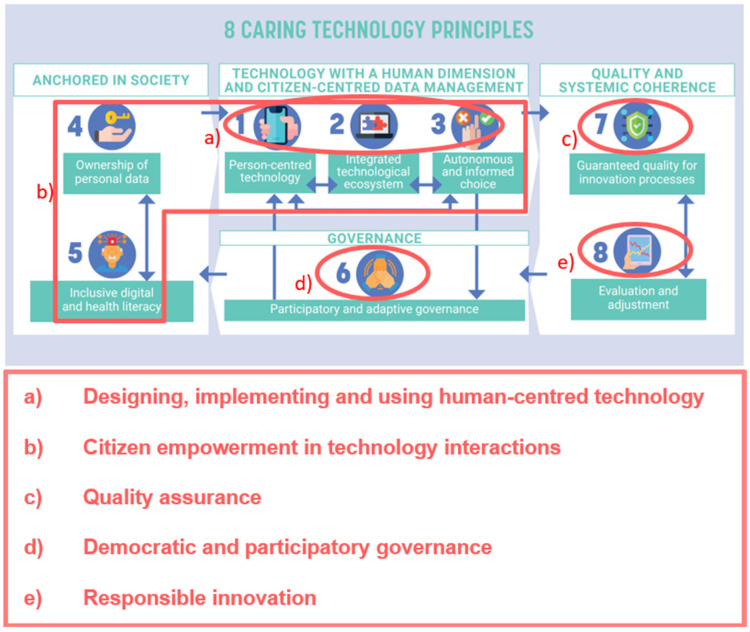
Mapping of the five thematic fields onto the 8 Caring Technology principles. Principles 1–3 relate to a) human-centred technology design, principles 1–5 to b) citizen empowerment, principle 6 to d) democratic and participatory governance, principle 7 to c) quality assurance, and principle 8 to e) responsible innovation.

To bridge this gap, the Dr. Daniël De Coninck Fund and the King Baudouin Foundation (Belgium) initiated a national co-creation process, resulting in the 8 Caring Technology Principles (CTP): a comprehensive ethical framework to develop, implement and evaluate caring healthcare technologies [23]. The Caring Technology Principles include (1) Person-centred technology, (2) Integrated technological ecosystem, (3) Autonomous and informed choice, (4) Ownership of personal data, (5) Inclusive digital and health literacy, (6) Participatory and adaptive governance, (7) Guaranteed quality for innovation processes, and (8) Evaluation and adjustment, and reflect a growing international call for ethical and human-centred approaches in digital health. To operationalise the principles and support their practical application, they were further structured into five thematic fields: (1) Designing, implementing and using human-centred technology, (2) Citizen empowerment in technology interactions, (3) Quality assurance, (4) Democratic and participatory governance and (5) Responsible innovation (Appendix A Table A1). The overlap between the Caring Technology Principles and the thematic fields is presented in Figure 1.

This paper explores how ethical frameworks can support the responsible development and implementation of caring technologies in older adult care as this population is at risk of being excluded from technological advances meant to support their health, independence, and societal participation. The study presents an exploratory pilot, offering an initial blueprint for a practical process guide by identifying the core needs and design requirements that should structure such a tool. The fully operational guide was developed in later stages of the EMPOWERCARE project and therefore falls outside the scope of this manuscript.

## 2. Materials and Methods

The research was performed in the context of the Interreg 2 Seas project EMPOWERCARE [39]. The project partners involved in this study included care organisations, research institutions, technology developers, and government bodies, spread across the Interreg 2 Seas regions of the United Kingdom (England, from Cornwall to Norfolk), the Netherlands (Zeeland and the coastal areas of Zuid-Holland, Noord-Holland, and Noord-Brabant), Belgium (Flanders), and France (Northern France, including Picardie). These partners conducted technology trials to evaluate the implementation and effectiveness of digital solutions in older people’s care. The trials, which involved individuals aged 65+ and individuals aged 50+ with at least one chronic condition, served as real-world use cases to reflect on and discuss the proposed ethical framework. An overview of the technology trials, including technology categories and types, is provided in the Appendix A (Table A2).

**Figure A1:**
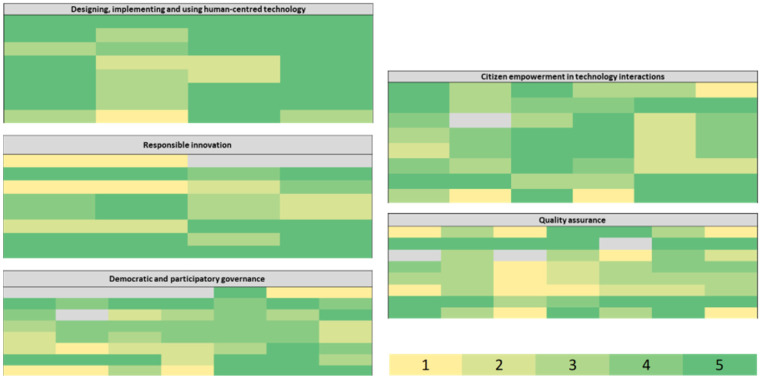
Relevance scores of the five thematic fields (questionnaire responses). The bottom-right bar shows the colour–score mapping. Grey indicates the statement was not applicable to the organisation.

Initial input was gathered through a questionnaire to assess stakeholder needs regarding the five ethical thematic fields of the CTP: (1) Human-centred technology design and implementation, (2) Citizen empowerment in technology interactions, (3) Quality assurance, (4) Democratic and participatory governance, and (5) Responsible innovation. Each thematic field comprised four to seven related statements, totaling 28 statements. Participants scored each statement on a 5-point Likert scale (1 = low relevance; 5 = high relevance) based on its relevance as a learning objective within their technology trial. This resulted in an overview of the relative relevance of the five thematic fields across partner organisations (Figure A1). While this input informed the design of the subsequent steps, the main findings presented in this paper are based on interview data collected with the project partners to identify key factors for the process guide.

The eight partner organisations that were running a technology trial were invited for in-depth interviews to discuss their questionnaire responses and reflect on practical considerations for implementing an ethical framework. Participants were purposively sampled based on their involvement in the technology trials to ensure sufficient depth and completeness of organisational perspectives. This resulted in a total of ten participants: five from care organisations, two from local governments, two from technology developers, and one from a research institution. A detailed overview of participants is provided in the Appendix A (Table A3). The semi-structured interviews were conducted online via Microsoft Teams. A structured interview guide, outlined in Appendix A (Table A4), was used to (1) examine regional implementation contexts, (2) explore needs and perspectives towards an ethical framework, (3) assess contributions from technology trials, and (4) identify essential considerations for a process guide.

Each interview lasted approximately one hour and was recorded and subsequently anonymised. Interview recordings were transcribed and thematically analysed using NVivo (Release 1.6.1, QSR International). An initial set of coding categories was developed through open coding, after which related codes were grouped and refined into higher-level categories consistent with axial coding. This iterative approach enabled systematic comparison across interviews and supported the development of coherent themes. Coding was conducted by one researcher, with iterative discussions among co-authors to ensure consistency and consensus. The final coding structure is presented in the Appendix A (Table A5). All findings were returned to participants for validation and feedback [40]. The interviews served to generate the empirical basis for a blueprint of an ethical process guide. Additional co-creation activities conducted later in the project (two external workshops and one focus group) further refined this blueprint, but the detailed development of the final guide lies outside the scope of the present manuscript.

## 3. Results

### 3.1. Regional Implementation

To investigate how ethical frameworks can inform the development and implementation of caring technologies, we first analysed the potential influence of regional context (Belgium, the UK, the Netherlands, and France) on both the application of ethical frameworks and the implementation process itself. Participants were asked if there was a certain regulation, context or macrosystem present that they needed to consider in their technology trial. Additionally, they were asked which of the five thematic fields of the CTP—(1) Human-centred technology design and implementation, (2) Citizen empowerment in technology interactions, (3) Quality assurance, (4) Democratic and participatory governance, and (5) Responsible innovation—were relevant or less relevant in their technology trial, taking into account the presence of certain regulations, contexts or macrosystems. Lastly, participants were asked whether those influenced the implementation of technology or the use of the thematic fields. Table 1 displays the description of the regulations, context or macrosystem, and the influence on the use of the thematic fields or technology implementation.

Overall, the regulations that participants were subject to could be divided into external and internal regulations. Participants followed external regulations—such as the General Data Protection Regulation (GDPR)—regulations from the government and ethical standards:

*PARTICIPANT 2 (Local government): ‘We have legislation called the Care Act. So, we have to make sure that we consider the Care Act and the requirements for that. Then there are technology standards that have to be considered for the technologies that we use. (…) We’ve had support (…) from technical experts who have been able to check that those solutions meet those standards.’*

Moreover, certain internal initiatives were present in the organisations, such as a data officer. A participant indicated to work with an eHealth platform which ensured quality, cybersecurity and interoperability when sharing the data with, for example, general practitioners, which corresponds to thematic field ‘Quality assurance’. As governance is structured in local authorities, one participant reported that it leads to focus on ‘Democratic and participatory governance’. Although there are regulations on the European, national and organisational level, participants indicated that these had little influence on the use of an ethical framework or implementation of technology in the different regions.

Overall, this suggests that while partners operated within a shared regulatory baseline (e.g., GDPR), the perceived ethical challenges were less driven by regional differences and more by organisational structures and responsibilities, highlighting the need for an implementation guide that is adaptable across contexts.

### 3.2. Needs and Perspectives Towards an Ethical Framework

To investigate in more detail the need and perspectives towards an ethical framework, participants were questioned about the relevance of the five thematic fields in their technology trial (Table 2).

Across interviews, a consistent pattern emerged: the first two thematic fields (human-centred technology and citizen empowerment) were seen as intrinsic to the project’s mission, whereas the remaining fields (quality assurance, governance, and responsible innovation) were often perceived as more abstract or located at higher decision-making levels.

‘*Designing, implementing and using human-centred technology*’ was ranked as most relevant. Overall, participants agreed on its importance due to the project’s focus on centralising the end-user in technology applications. Participants were already familiar with the topic and implementing the concept in their technology trials:

*PARTICIPANT 4 (research organisation): ‘I think it is the main philosophy of the EMPOWERCARE project. So, if we go a few steps back, people that wanted to engage with the EMPOWERCARE project, they had this as a philosophy and mentality, hence it scores I guess so high.’*

One participant indicated that technology developers do not pay enough attention to this thematic field.

The thematic field *‘Citizen empowerment in technology interactions’* was also considered relevant, with five out of ten participants rating it as such. They noted that this theme aligned with the project’s broader focus on promoting active involvement of end-users in digital care solutions.

*PARTICIPANT 6 (care organisation): ‘When it comes to empowerment, that development and interaction start from the people themselves, it is logical that this is the most important thing within EMPOWERCARE. […] EMPOWERCARE itself also strongly steers towards those first two thematic fields. (translated)*

Compared to the first two thematic fields, participants perceived ‘Quality assurance’ as less directly relevant to their work. During the interviews, several participants explicitly described this domain as outside their expertise and primarily the responsibility of developers or internal specialists. They emphasised the need for greater attention to this topic within their organisations. One participant highlighted the importance of acknowledging the responsibility of quality assurance, even without being an expert:

*PARTICIPANT 2 (local government): ‘I think it’s just about knowing that you need to pay attention to these elements, but also you need to throw in your experts within your organization to support those areas.’*

Another participant indicated that this field is more subject to decision-makers:

*PARTICIPANT 5 (care organisation): ‘That is not to say that the operational partners, the people who run the project, are less interested, but they are much more subject to the decisions of the decision-makers.’ (translated)*

In addition, participants reported the necessity for decision-makers to work bottom-up instead of top-down. This reflects a broader tension reported by several partners: ethical aspirations were recognised, but responsibility for acting on them was frequently attributed to external experts or higher-level governance structures rather than operational trial teams.

For the fourth thematic field, *‘Democratic and participatory governance’*, participants expressed mixed views on its relevance compared to the first two thematic fields, though more of them could connect this field to their organisational context than was the case for *‘Quality assurance’*. Some had already addressed elements of this theme in their technology trials, such as tackling potential inequalities or including stakeholder perspectives. For local authority partners, aspects like accountability and responsiveness were seen as particularly important—especially given their responsibility toward vulnerable populations and their role in ensuring fair and transparent public service delivery. As one participant put it:

*PARTICIPANT 3 (local government): ‘Accountability and responsiveness are always important for local authorities […] Our target group are mainly vulnerable people who need help of public policies.’*

For the final thematic field, *‘Responsible innovation’*, participants felt again that too much attention was placed on human-centeredness, while responsible innovation deserved greater focus. Many saw the topic as beyond their direct influence and more relevant to technology developers, assuming it was already addressed through existing standards and regulations. Operational partners involved in the pilots also noted that decisions regarding responsible innovations were often made higher up, emphasising the need for a bottom-up rather than top-down approach.

*PARTICIPANT 1 (care organisation): ‘I think this plays out more at a higher level, and as a small organisation we have little direct involvement with it, precisely because it takes place above us. […] These themes are quite abstract and less immediately relatable than, for example, human-centred design.’ (translated)*

Taken together, these findings indicate that partners valued the full ethical scope of the CTP, but experienced an imbalance in practice, with stronger engagement in user-facing domains and more limited ownership of system-level responsibilities. This imbalance directly informed the blueprint to support organisations beyond the design phase alone.

### 3.3. Contributions from Technology Trials

To explore how implemented or completed technology trials could inform a process guide, participants were asked to share good practices from their experiences. Good practices were most concrete in thematic fields directly connected to daily care practices (e.g., human-centred design and empowerment), whereas system-oriented fields such as quality assurance and responsible innovation yielded fewer operational examples. Table A6 includes the full list of responses that were given by the participants for each of the CTP.

Designing, implementing and using human-centred technology

Several good practices focused on involving end-users throughout the process. Examples included intake conversations and follow-up meetings with end-users and their families to gather feedback, and a co-creation approach spanning the full design phase. A community-based approach was also highlighted, in which local networks supported older adults rather than relying solely on professional care workers. To build an integrated technological ecosystem, participants stressed the importance of addressing interoperability issues by testing technologies directly in end-users’ homes, given the variation in devices and setups. One participant emphasised only working with trusted partners already active in the ecosystem. Ensuring informed and autonomous choices was supported through actions like providing clear information leaflets for family members and offering live, in-home demonstrations in the user’s own language.

Citizen empowerment in technology interactions

Respecting institutional ethics frameworks around data sharing and privacy was seen as key to protecting end-users and safeguarding their ownership of personal data. Multiple good practices aimed at enhancing digital and health literacy were reported, such as training volunteers to act as *Digital Ambassadors*:

*PARTICIPANT 2 (local government): ‘[We ensure] that they have training around how they can then be Digital Ambassadors, (…). So how do they work with older people? How do older people retain information? How do they like to engage? We have commission training for the Digital Ambassadors (…), so that they feel confident in their role as a Digital Ambassador, so they can go work with people.’*

To reach people in vulnerable situations, some participants negotiated lower prices or bundled service packages with technology suppliers. Others referred users to social services for financial support and accounted for costs from the start to avoid unexpected burdens for end-users.

Quality assurance

To ensure quality in innovation processes, participants recommended starting with small-scale technology trials before scaling up to broader organisational implementation.

Democratic and participatory governance

Democratic involvement was supported through mechanisms like resident councils and family councils, allowing end-users and their families to actively participate in decisions.

Responsible innovation

Responsible innovation was supported through regular meetings involving care managers, innovation officers, and relevant departments to evaluate both the technology and its implementation, and to make adjustments where needed. Safeguarding end-users was also seen as essential. Participants stressed the importance of acting as a liaison: ensuring that technology companies cannot approach end-users directly without prior review and oversight. One participant also reported adhering to the ethical standards set by their own organisation.

Overall, the distribution of good practices illustrates how ethical implementation is often strongest at the interpersonal level, while organisational and governance-related principles require additional structural support; one of the core motivations for developing the proposed process guide.

### 3.4. Key Considerations for a Process Guide

To identify key aspects for inclusion in a process guide, participants were asked about unmet needs addressed by their technology trials, the types of support a potential guide could offer, and how they envisioned using it in practice. Table 3 shows the full list of responses of the participants.

Across responses, participants consistently expressed the need to translate ethical principles into actionable decision support, suggesting that the blueprint should function less as a normative framework and more as a practical implementation tool.

Participants were first asked to describe the specific needs their technology trials aimed to address. Since the trials targeted individuals aged 50 and over with at least one chronic condition, most unmet needs focused on supporting older adults. Half of the participants indicated that improving digital literacy among both seniors and care staff was a core objective. Others focused on empowering older adults, strengthening their social networks, and providing adequate care or remote activities, especially in response to the COVID-19 pandemic.

To understand how a potential process guide could respond to these unmet needs, participants were asked to provide possible solutions. Overall, participants struggled with the theoretical nature of the ethical framework. Therefore, they expressed the need for concrete tools and examples—both good and bad practices—linked to the thematic domains. Particularly in relation to teaching seniors and workforce to deal with modern technology and providing sufficient care, some participants noted that the acceptance of technology was often more difficult for care staff than for older adults:

*PARTICIPANT 2 (local government): ‘I found that the end-users are quite open to these solutions [technologies], but what we find is the challenge around our workforce promoting and encouraging people to look at technology.’*

*PARTICIPANT 1 (care organisation): ‘Care workers very often have a tendency to take over. Instead of empowering the older person, they just say: ah, you can’t do it anymore, I’ll do it for you.’ (translated).*

Participants therefore stressed the importance of including guidance on how to engage and support care professionals in adopting new technologies.

When asked how they envisioned using a process guide in practice, participants generally viewed a process guide as a potential quality label—a tool to signal that technologies aligned with the guide’s principles met certain standards:

*PARTICIPANT 10 (technology development): ‘I think that is how we could use such a [guideline], that it really is a kind of quality label, which we can apply ourselves to our own technologies and also communicate to the partners with whom we work.’ (translated)*

The guide was also seen as a means to support more objective decision-making when selecting technologies or projects:

*PARTICIPANT 6 (care organisation): ‘The intention, however, is really to use [the guideline] to support decision-making: which products and which products not, which projects yes and which projects no.’ (translated)*

Finally, participants noted the importance of using a process guide early in the development process, to integrate ethical considerations from the start. One participant emphasised its value in showing stakeholders the relevance of caring technology:

*PARTICIPANT 10 (technology development): ‘(…) that we can actually demonstrate to all those stakeholders that there is significant added value in using the guideline.’ (translated)*

## 4. Discussion

This study explored how ethical frameworks can support the responsible implementation of caring technologies, using the Caring Technology Principles (CTP) as conceptual basis and older adult care as an initial case. To achieve this, in-depth interviews were conducted with representatives from partner organisations of the EMPOWERCARE project in the four participating countries: the UK, the Netherlands, Belgium and France. These interviews aimed to map any existing regulations or frameworks in those countries, assess the fit of the CTP for their needs, and explore how it could be translated into a practical process guide for technological innovation.

Findings showed that all investigated regions complied with their respective national regulations, which varied by country. Nevertheless, medical technologies within the European Union adhere to uniform, stringent requirements for obtaining the CE mark [29,41,42]. However, in the United Kingdom, the UK Conformity Assessed (UKCA) label replaced the CE mark for medical technologies [43] following Brexit. Despite the variations in national regulations, results illustrated that ethical considerations were largely shared across the participating countries and that they did not significantly influence the implementation of technology. European frameworks emphasise the importance of innovation in healthcare, but there is still limited enforcement of innovation-driven policy measures, suggesting that further work is needed to embed any framework into routine regulatory practice. This lack of practical guidance and enforcement underscores the broader transferability and relevance of ethical frameworks designed to guide day-to-day decision-making in technological innovation, such as the Caring Technology Principles [23].

Furthermore, our study identified a gap in current regulations in the four countries to promote digital literacy and inclusion for technology, despite its increasing relevance in healthcare and public mental health promotion [31]. Indeed, participants highlighted the importance of centralising end-users in technology implementation and enhancing digital literacy, aligning with global trends toward human-centred technology design [44,45,46]. They confirmed a strong need in these technologies to empower older adults in their healthcare management and support digital accessibility, not only for the end users but also for the care workers and professionals.

Quality assurance, democratic governance, and responsible innovation, on the other hand, received less emphasis from the participants, often being perceived as abstract, externally driven, or beyond the immediate influence of project implementers. A broader policy comparison indeed suggests that quality assurance is already highly regulated, but responsible innovation remains more of a policy goal than a regulatory standard [31]. The participants perceived that these aspects were beyond their direct influence or that these decisions were made ‘higher up’ in the healthcare system. At the same time, they expressed a clear preference for more bottom-up approaches in these matters, with active involvement of care workers and local stakeholders in shaping ethical decisions—rather than having them imposed from higher levels of governance. This aligns with recent literature emphasising that ethical frameworks gain legitimacy and practical value when shaped through lived experiences and local engagement. Rather than imposing abstract principles from above, ethics in technology implementation should emerge collaboratively from within care settings, reflecting the values of professionals, users, and communities involved [47,48].

Furthermore, some professionals saw themselves as ethical gatekeepers—liaisons who actively safeguarded end-users by mediating contact with technology providers and ensuring ethical standards were upheld. This highlights an emerging professional role within care ecosystems: one that is grounded in proximity to the citizen while navigating the ethical challenges of digital transformation. Recent research confirms that professionals actively negotiate the ethical use of technology, maintaining discretion to reject or adapt its use when it risks undermining patients’ well-being and relational autonomy [49]. Notably, some participants mentioned trained Digital Ambassadors to act as intermediaries between technologies and less digitally skilled users. These volunteer roles closely resemble link workers or community health workers, who act as trusted figures embedded in local communities and who are increasingly recognised as pivotal in ensuring ethical, context-sensitive support for vulnerable populations [50,51,52]. They combine technological guidance with a broader social support function, making them well-positioned to bridge the gap between system-level innovation and individual needs. While the COVID-19 pandemic accelerated digital adoption, further efforts are needed to integrate technology seamlessly into care delivery [53].

When asked about concrete needs, starting from the technology trials partners were implementing in older adult care, it became clear that although the guiding principles and themes in the CTP suited the needs of the participants, some experienced difficulty in translating an ethical framework into practice. While policymakers and innovation managers found it easier to apply the principles, care providers struggled with their theoretical nature. They expressed the benefits of translation into a practical, structured process guide to support decision-making and implementation.

The study identified innovation managers as the primary target group for a process guide, given their decision-making role in implementing new technologies. A key issue identified was that technology adoption often requires significant workflow adjustments, and without clear implementation guidelines, professionals felt unprepared to integrate new technologies effectively. Research indicates that process guides enhance decision-making transparency and help organisations systematically evaluate healthcare technologies [54,55]. Incorporating design thinking methods could further improve usability and encourage iterative development [56]. Participants also stressed that care providers, procurement officers, and end-users must be actively involved as well to ensure a process guide reflects real-world challenges. A bottom-up approach is essential to successful adoption. An important insight from the trials they were conducting at the time of the study was that technology resistance was not limited to end-users; healthcare professionals also expressed reluctance due to concerns over job displacement and workflow disruption. It also aligns with broader evidence showing that cross-sector collaboration—between healthcare professionals, policymakers, developers, and users—leads to more successful technology integration [57,58,59,60]. Involving staff in decision-making can improve motivation, reduce burnout, and help address ongoing workforce shortages in healthcare [2,16,17,18,61,62]. To support this, a process guide should suggest structured workshop formats for innovation managers. These workshops are designed to involve care staff and end-users in the early stages of implementation, allowing their needs and expectations to inform the process.

Participants envisioned using a process guide as a quality label—a recognisable standard to assess whether technologies align with ethical and user-centred principles. This labelling function can help organisations not only select appropriate technologies, but also communicate shared values and ethical commitments to partners, funders, and end-users. To maximise its impact, participants emphasised that the guide should be introduced early in the development cycle, allowing ethical considerations to shape choices from the outset rather than being retrofitted later on. This aligns with emerging international efforts to formalise ethical values through process-based frameworks and certification models—such as the Z-Inspection^®^ process and the Assessment List for Trustworthy AI (ALTAI), specifically for AI solutions [63,64].

Participants highlighted the need to include good practices to make an ethical framework more practical. Organisations like the OECD promote innovation through curated good practices, and Keppell et al. stress that not sharing such practices can slow progress [65,66]. Existing healthcare initiatives already collect and share good practices [67,68]. A process guide should offer a structured way to apply them. To remain relevant, the list of good practices must be regularly updated. Embedding a process guide in a network would support ongoing improvement and ensure sustainability beyond the project. Finally, to ensure and facilitate local compliance, participants expressed the usefulness of including an overview of relevant regulations in a process guide.

The final version of a process guide should address these reported challenges and opportunities. By offering a step-by-step approach, a process guide will enable more objective decision-making when assessing technology quality, making an ethical framework more actionable. Additionally, documented good practices from existing initiatives can provide insights into overcoming digital literacy barriers, affordability concerns, and resistance to innovation. Finally, a process guide should encourage organising workshops with management, the workforce, and end-users, thereby facilitating idea implementation, bridging the gap between end-users and developers, and enhancing technology integration within organisations and care environments. A process guide should therefore offer not just a synthesis of ethical principles, but a usable and actionable guide tailored to the complexity of care innovation, ideally introduced early in the process to guide key decisions from the start.

This study holds a few limitations. First of all, this is an exploratory pilot and is based on a small, purposive sample of ten participants, which is common in the initial phase of qualitative, in-depth research. While thematic saturation was approached for the scope of this study, the sample size remains limited. The stratification across countries, organisational types, and technology use cases was intentional, as this phase aimed to capture diverse perspectives to inform the initial blueprint for a process guide. A second limitation is that the study presents the blueprint rather than the fully developed guide; its operationalisation was undertaken in later phases of the EMPOWERCARE project, including a broader set of stakeholders. Another limitation of this study was that end-users, being older adults, were not directly involved, though their perspectives were captured through care organisations. As the guide is designed for innovation managers and developers, this choice was methodologically intentional. However, future research should evaluate how the guide supports meaningful end-user involvement in practice and how this influences ethical technology implementation. Future research should validate the guide with larger and more varied stakeholder groups, refine participatory models, and examine how regional contexts and care settings influence long-term adoption and scalability. Exploring how ethical frameworks can be embedded into formal evaluation procedures, such as adapted HTA models, would strengthen the integration of ethics into innovation governance.

## 5. Conclusions

This study explored how ethical frameworks can support the responsible development and implementation of caring technologies in older adult care. This resulted in the first outline of a practical process guide to support the implementation of caring technologies, using ageing societies as an initial context and the CTP as conceptual scaffolding. Key priorities included centring end-users and addressing digital literacy, while themes like responsible innovation and quality assurance proved harder to operationalize. A core challenge was the limited applicability of abstract ethical principles in daily care settings. While existing initiatives often define ethical domains, few offer a structured, actionable process to guide implementation in practice. The proposed guide responds with a step-by-step structure, practical examples, and participatory tools to support inclusive, value-driven technology adoption. It is envisioned both as an implementation aid and a quality label to align stakeholders. Future research should validate the guide’s usability, explore its role across care contexts, and examine how ethics can be more firmly embedded in innovation governance.

## Figures and Tables

**Table 1:** Regional regulations, contextual factors, and their influence on technology implementation.

Description Regulations, Context or Macrosystem	Influence on Use of Thematic Fields or Technology Implementation
General Data Protection Regulation (GDPR) (2)	Governance structured in working local authorities, thus attention paid to ‘Democratic and participatory governance’
Laws and regulations	Legislation important for procurements
Rigid regulations from government	Already focus on quality
Legislation Care Act	Working with eHealth platform to ensure quality, cybersecurity and interoperability when sharing data with, e.g., general practitioners
Technology standards	
Data protection policies	
Ethical standards	
Regulations within organisation	
Data officer within organisation	
Financed by government, thus accountability	
Contract with end-users, no other difficulties	
Open policy	
No collection of personal sensitive data, thus no concerns intruding people’s lives	

**Table 2:** The relevance of and needs towards the thematic fields.

Thematic Field	Relevance and Needs
Designing, implementing and using human-centred technology	Already focus of attention in project (7) Implicit focus of attention in technology trial Less interest by partners in technical aspect of technology trial Scoring high because first step in development More relevant for higher level Not enough time and money in this stage of development Operational partners carrying out the technology trial are subject to decision-makers Technology implementers have less control over it Operational partners carrying out the technology trial are subject to decision-makers Already used to working with it
Citizen empowerment in technology interactions	Already focus of attention in project (5) Not enough attention paid (2) Stakeholder’s aspect very relevant in technology trial Addressing potential inequalities very relevant in technology trial Technology developers do not pay enough attention
Quality assurance	No technology experts (2) Not enough attention paid More relevant for technology developers Necessary to move focus of attention from first two thematic fields (Designing, implementing and using human-centred technology & Citizen empowerment in technology interactions) to others
Democratic and participatory governance	Very relevant Relevant when public and private sector cooperate Accountability and responsiveness relevant for local authorities Operational partners carrying out the pilot are subject to decision-makers Necessary to work bottom-up instead of top-down
Responsible innovation	Common agreement on importance Should be very relevant in all technology trials, especially scaling up Scoring high because ‘expected’ in project Technology developers do not pay enough attention

**Table 3:** Identified unmet needs, support and eventual use of the process guide.

Current Unfulfilled Needs of Technology Trials	Types of Support of the Process Guide	Eventual Use of the Process Guide
Teaching seniors and workforce to deal with modern technology (5)	Encouraging and teaching workforce to use technology (4)	Quality label (6)
Providing sufficient care (3)	Making the principles practical (3)	Facilitation of objective decision-making (2)
Technology affordability (2)	Good and bad practices (2)	Guidance for technology (implementation) (2)
Providing care or activities from a distance (2)	List of relevant, already existing networks (2)	Before starting technology trial already considering principles
Empowerment of senior (2)	Making a technology sustainable (2)	Showing relevance of creating a caring technology
Supporting seniors and their social network	Organ checking technologies and approving them	Encouraging workforce to use technology
Providing simple technologies	Continuation once product is on the market	Use in broader context
Encouraging ‘technology first’	Points of growth of other technology trials	
Making care attractive	Overview of regulations, licences and checklists	
Gaining control over data		
Sustainable reuse of data		

## Data Availability

The raw data supporting the conclusions of this article will be made available by the authors on request.

## Abbreviations

The following abbreviations are used in this manuscript:

AI	Artificial Intelligence
ALTAI	Assessment List for Trustworthy AI
CTP	Caring Technology Principles
CE	Conformité Européenne
EU	European Union
GDPR	General Data Protection Regulation
HTA	Health Technology Assessment
eTA	ethical Technology Assessment
VSD	Value-Sensitive Design
RRI	Responsible Research and Innovation
UK	United Kingdom
UKCA	United Kingdom Conformity Assessed
UZA	Universitair Ziekenhuis Antwerpen
WHO	World Health Organization
OECD	Organisation for Economic Co-operation and Development

## Appendix A

**Table A1:** The five thematic fields based on the Caring Technology Principles.

Designing, implementing and using human-centred technology	Target audience(s) and what they are trying to achieve relevant to the service you try to provide
	Problems that the target audience(s) experience in the current situation (before the tech trial)
	Possible solutions for better achieving the goals of the target audience(s) (besides the technological solution that you want to test)
	Experiences of the target audience(s) in using the technology
Citizen empowerment in technology interactions	Protection of privacy (e.g., harvesting people’s personal data cannot be considered the unique selling proposition of technology)
	Enabling autonomous choices on sharing data where possible
	Increasing citizens’ opportunities for undertaking action to improve health and wellbeing (based on sharing insights gained through the technology trial)
	Strengthening digital literacy
	Strengthening health competences
	Giving special attention to vulnerable groups (e.g., by lowering participation thresholds)
Quality assurance	Accuracy of data collection
	Representativeness of data collection (i.e., are your data representative of a wider possible audience)
	Proportionality of data collection (i.e., are you collecting just the data you need for your service)
	Cybersecurity
	Transparency (i.e., providing open and accessible information about the innovation process and its purposes)
	Effectiveness (i.e., is the project achieving its goals?)
	Interoperability (i.e., ensuring that data can be used by other relevant applications)
Democratic and participatory governance	Engaging citizens and stakeholder groups in setting the basic parameters of governance
	Enabling a wider dialogue and deliberation with stakeholders and citizens outside of the immediately involved on the vision, purpose and goal of the technology
	Accountability and responsiveness (e.g., adapting to changing circumstances, mechanisms of redress if something goes wrong)
	Ensuring independent oversight (‘checks and balances’)
	Ensuring fairness of the outcomes of technology implementation
	Collaboration with trusted partners
	Addressing potential inequalities (i.e., ensuring that your technology also addresses the needs of typically disadvantaged groups)
Responsible innovation	Complying with the law
	Complying with ethical standards (e.g., reviews by ethics committee)
	Anticipation of the wider consequences of technology implementation when scaling up
	Reflectiveness (periodic reflection on the question whether the way the technology is being embedded in society is still respecting the original principles of caring technology)

**Table A2:** Overview of the technology trials conducted by the partner organisations.

EMPOWERCARE Partner Organisation	Technology Category (AT *—ICT **—HCI ***)	Technology Type
1	AT	Toolbox with different devices
2	ICT HCI	Tablets Voice activated technology
3	ICT	App
4	ICT	Video calling with TV
5	ICT	Video calling with tablet
6	AT	Room with different devices
7	ICT	Video calling in group
8	ICT	Online health platform

* AT: Assistive Technologies; ** ICT: Information and Communication Technologies; *** HCI: Human–Computer Interaction technologies.

**Table A3:** Overview of the participants in the interviews.

Organisations and Their Participants		
Care organisation	5	Participant 1: Care organisation (BE)—occupational therapist/movement consultant; Participant 5: Home Care organisation (BE)—(1) care policy manager and Participant 6: (2) care policy product manager (policy and innovation); Participants 8: Social Care organisation (NL)—(1) policy advisor and Participant 9: (2) strategic advisor, interim and programme manager
(Local) government	2	Participant 3: Regional Government Body (FR)—project officer; Participant 2: County-level government (UK)—project manager
Research organisation	1	Participant 4: University (UK)—principal lecturer
Technology developer	2	Participant 7: Technology Company (BE) managing director; Participant 10: Research & Technology Institute (BE) R&D
Total	10	

**Table A4:** The interview guide.

**Research Questions**
1. **How** are the Caring Technology Principles relevant for or used by the different stakeholders?
2. What are important aspects that should be considered or added to the Caring Technology guideline, taking into account different stakeholders?
3. How can implemented or completed technology trials contribute to the Caring Technology guideline?
4. How does the region of technology implementation influence the use of principles or implementation of the technology?
**Interview Questions**
1. Would you like to introduce yourself and your organisation? a. What is your specific role within the EMPOWERCARE project? b. Can you briefly explain what your technology trial entails? i. How did this technology trial come about? ii. What unmet needs does your technology trial aim at? iii. Why did you choose this specific technology trial? iv. What stage is your technology currently in? Is it possible to already evaluate the thematic fields against the actual implementation? If no, when would you be able to do so?
2. You have already filled in the intake form and given scores to the different subcategories of the thematic fields; how did the scoring go? Did you experience any difficulties/problems with certain subcategories or thematic fields?
3. Is there a certain regulation, context, or macrosystem present that you need to consider in your tech trial?
4. Taking into account the presence of certain regulations, a certain context, or a macrosystem, have you already used certain thematic fields during your technology trial? a. Which thematic fields are relevant in your technology trial? b. Which thematic fields are less relevant in your technology trial? c. Do the regulations, context, or macrosystem influence the implementation of technology or the use of thematic fields?
5. Thematic fields ‘Designing, implementing and using human-centred technology’ and ‘Citizen empowerment in technology interactions’ overall score high. What are your thoughts on this? a. How are these subjects (more) relevant? b. How do you implement these fields in your technology trial and how can these experiences be a contribution to the guideline?
6. Thematic fields ‘Responsible innovation’ and ‘Quality assurance’ overall score lower than other thematic fields. What are your thoughts on this? a. Do you think these subjects are not relevant to EMPOWERCARE partners? Do you think that the guideline should focus more on these subjects in order to guide partners towards more responsible innovations for which quality is assured?
7. Could you describe in just a few sentences in what way the guideline would be a clear added value for your organization? a. What should be added to the guideline? b. How will you use the guideline?

**Table A5:** The interview codebook.

Type of organisation
Role within EMPOWERCARE
Technology trial
Description
Unfulfilled need
Current phase
Filling in intake form
Regulation, context, macrosystem
Description
Influence on the thematic fields
Thematic fields
Designing, implementing and using human-centred technology
Citizen empowerment
Quality assurance
Democratic and participatory governance
Responsible innovation
Others
Guideline
What should be added
How used
Good practices
Designing, implementing and using human-centred technology
Citizen empowerment
Quality assurance
Democratic and participatory governance
Responsible innovation
Remarks

**Table A6:** Good practices from implemented technology trials.

1. Person-centred technology	Intake conversations and intermediate meetings with end-users and family
	Involving service provider to learn about needs of end-users
	Involving family, friends, neighbours when accessibility problems for end-users arise
	Allowing end-users to work with technology during user interface sessions and choosing which version works best
	Involving end-users when developing a service for a product, questioning and representing the end-user with regard to the supplier when the product is further developed
	Cooperation from end-user to research institution, e.g., during design
	Open field training of the workforce on the technology trial site
	Community-approach to help seniors instead of counting only on professional workforce
2. Integrated technological ecosystem	Testing and evaluating technology in end-user’s own homes to explore issues of interoperability due to variation of devices and required connections in each different home
	Working only with trustworthy partners
	Bringing together different partners
	Cooperation between partners ensures data can be used by other relevant applications
3. Autonomous and informed choice	Information leaflet for family
	Online tutorials on how to work with technology
	Permission of end-users to be in photographs
	No additional informed consent, unless person wishes to participate in a specific project, e.g., EMPOWERCARE project
	Organising coproduction workshops with end-users before proceeding with trials to explain different types of technologies, sessions give opportunity to end-users to ask questions and resolve concerns
	Trials and data collection always approved by ethics committee to provide additional layer of scrutiny and transparency
	Working with informed consent
	Direct contact information of technology provider for end-user
	Live demonstration and installation at end-user’s home, ‘in the end-user’s language’
4. Ownership of personal data	Coproduction workshops contribute to building trust
	Adhering to ethics framework of institution regarding data sharing and privacy to ensure any activity is safeguarded for the end-user
	Paying attention to cybersecurity by detecting end-users via sensors without camera footage/identity information
	Data collection on the field instead of reporting afterwards
	Consider a broad range of relevant data source (while adhering to data minimization)
	Connection with eHealth platform
5. Inclusive digital and health literacy	Using trained volunteers to help people improve end-user’s digital literacy
	Online/paper tutorials on how to work with one specific technology
	Communication system about end-users between workforce to optimise cooperation/personalised care
	Communication via Facebook page to family and volunteers
	Communication via WhatsApp groups for workforce
	After trying out technology, end-user is financially supported for a buying option
	Offering voluntary support for technical issues and answering questions when conducting workshops
	Sharing list of supporting organisations and online sources with end-users
	Convincing governments to set up 100 centra where people can improve their digital literacy
	Taking costs (that are otherwise for end-users) into account at start project
	Bringing end-users in contact with social services that can help financially
	Negotiating prices and complete service packages for the customer with suppliers
	Visualising the full price tag
	Asking suppliers to provide sufficient low-threshold communication and manuals in various formats, e.g., video, paper
	Open field training of workforce on technology trial site
6. Participatory and adaptive governance	Residents’ councils and family councils
	Good partnerships with local councils and learning/being in line with their governance
	Stakeholder analysis
	Involving and questioning end-users in workshops, e.g., development of planning app for employees
	Broader network for discussions, outside of directly involved partners
7. Guaranteed quality for innovation processes	Testing in small technology trial before rolling it out in a larger part of the organisation
	Evaluations during testing are carried out at predetermined times, usually based on a report
	Experimenting on a small scale with end-users and workforce to see what works and what does not
	Starting with technology that already has a basis
	Working only with trustworthy partners
	Trying out technology from technology developers before offering to end-users
8. Evaluation and adjustment	Organising meetings on a regular basis between care managers, innovation officers and department
	Looking what is already there, how to innovate within existing processes, existing methodologies are examined and deepened
	Contracts with technology suppliers to protect end-users
	Development of a website that meets objectives of a social support method and meets moral ethics
	Adhering to ethical standards
	During technology trials with industrial partners, always safeguarding end-users by acting as liaison, i.e., technology companies cannot approach end-user without review and scrutiny
